# Comparing the temporal dynamics of thematic and taxonomic processing using event-related potentials

**DOI:** 10.1371/journal.pone.0189362

**Published:** 2017-12-13

**Authors:** Olivera Savic, Andrej M. Savic, Vanja Kovic

**Affiliations:** 1 Laboratory for Neurocognition and Applied Cognition, Department of Psychology, Faculty of Philosophy, University of Belgrade, Belgrade, Serbia; 2 School of Electrical Engineering, University of Belgrade, Belgrade, Serbia; University of Akron, UNITED STATES

## Abstract

We report the results of a study comparing the temporal dynamics of thematic and taxonomic knowledge activation in a picture-word priming paradigm using event-related potentials. Although we found no behavioral differences between thematic and taxonomic processing, ERP data revealed distinct patterns of N400 and P600 amplitude modulation for thematic and taxonomic priming. Thematically related target stimuli elicited less negativity than taxonomic targets between 280–460 ms after stimulus onset, suggesting easier semantic processing of thematic than taxonomic relationships. Moreover, P600 mean amplitude was significantly increased for taxonomic targets between 520–600 ms, consistent with a greater need for stimulus reevaluation in that condition. These results offer novel evidence in favor of a dissociation between thematic and taxonomic thinking in the early phases of conceptual evaluation.

## Introduction

The speed and ease with which we recognize visual objects and sounds from our environment is truly impressive, making the question of the principles of organization of such efficient knowledge storage one of the most inspiring in psychology and beyond. In order to understand how the brain constructs meaning, we need to address the matters of content and timing of semantic processing. First, we need to understand what information regarding an object is available when a concept is activated, and second, which kind of information about an object is activated first, or what type of information is the most salient.

Meyer and Schvaneveldt [1] were the first to recognize the unique opportunity the semantic priming paradigm offers in uncovering the principles underlying conceptual organization. The mere presence of the word *cat* facilitates recognition of the word *dog*, which suggests that overlapping semantic information between these two concepts is available when the semantic representation of cat is activated. However, two objects rarely share only one type of information. *Dog* may be facilitated by *cat* because they share many features, they are members of the same semantic category, because dogs chase cats, or because words *cat* and *dog* frequently co-occur in language. Coexistence of different types of relations makes it difficult to untangle the specific effects different types of knowledge have on semantic processing.

The first attempt to disentangle the nature of the information supporting semantic priming was made by Fischler [2], who made the distinction between associative and semantic (non-associated, categorically related) primes, and showed that both types of information can be spontaneously accessed. In the research that followed, which aimed to differentiate between associative and semantic priming effects [3–4], conflicting patterns of results raised concern that different relationship subtypes may lead to different priming effects and thus suggested that it is important to disentangle the effects of different types of semantic information. In a meta-analytic study of semantic priming, Hutchinson [3] supports this argument by giving the example of the study of Moss and associates [5] in which three types of semantic information, co-ordinates (e.g. *pig*—*horse*), scripts (e.g. *restaurant*—*wine*) and instrument relations (e.g. *broom*—*floor*), were compared while controlling for strength of associative relatedness. Moss and associates [5] found the most robust priming effects for instrument related primes, and concluded that their results suggest that different types of semantic information may be activated with different time courses, which may explain different patterns of facilitation depending on the experimental design (modality and the prime-target delay). In other words, they found evidence that both (a) information regarding a dog’s features and the superordinate category it belongs to and (b) information regarding objects that frequently interact with a dog are spontaneously activated when the concept of dog comes to one’s mind. Their study even went a step further, suggesting that information regarding the function and situation seems to be activated more quickly than information regarding category membership.

In the study conducted by Moss et al. [5], the item pairs they refer to as ‘category coordinates’ (e.g. *pig*—*horse*) closely resembled what is typically denoted as taxonomic relations, relations between objects that belong to the same semantic category based on the overlap in features or meaning (*cow*–*donkey*, *apple–pear*)[6–7]. On the other hand, instrument relations in their study fit the definition of thematically related objects, objects that are related based on the complementary roles they play in the same scenario or event (*cow–milk*, *nail—hammer*) [7].

More recent studies on differences in taxonomic and thematic processing lend further support to the idea that the processing of thematic relations can be dissociated from the processing of taxonomic relations, in terms of the temporal dynamics, salience and ease of activation of the two types of information [7–9]. Thematic relations are found to be identified faster in explicit forced-choice tasks [10–11] and in the tasks where explicit judgment of the semantic relations is not required [12–13]. Eye movement patterns in the visual-world paradigm [12–13] show earlier and more transient semantic competition between the target and thematic distracters, while competition effects of distracters that share a taxonomic relation with the target (general and specific function) are detected later and are longer-lasting (for conflicting results see [14]).

Another way to track the time course of knowledge activation is to use techniques with high-temporal resolution, such as event-related potentials. Analogous to the way facilitation by congruent context shortens response latencies, congruent context leads to the reduction of the ERP wave amplitude in a time window between 250 and 550 ms after the target is presented—the N400 effect [15–16]. The reduction in amplitude of the N400 is interpreted as reflecting the easier semantic integration of the target and its context [15].

ERP studies designed to test differences between thematic and taxonomic information processing have yielded inconsistent results. Most studies have failed to find differences between thematic and taxonomic processing where they were most expected—in the N400 time-window [17–20]. In a recent study, Chen et al [20] suggested that thematic and taxonomic processing do not differ at the initial stage of processing (N400: 300–400 ms) when the general relatedness between the items is calculated, but that the dissociation happens at a later stage (P600: 500–600 ms) when, according to these authors, integration processes take place–P600 was larger for taxonomically related compared to unrelated and thematically related words. On the other hand, Chen et al [21] reported attenuated frontal negativity (400–550 ms) elicited by productive relations (*bee*–*honey*) compared to hierarchical relations *(offspring*–*grandson*), internal features (*gold–golden*), script relations (room–tenant) and unrelated (*star–spoon*) trials. Wamain and associates [22] failed to detect N400 effect differences in a naming task in which interstimulus interval between prime and target was 550 ms. However, using a similar design they observed attenuated N400 for thematic relations compared to categorical (general function) information when the interstimulus interval was shorter (366 ms). They interpreted these differences as demonstrating that taxonomic compared to thematic information takes more time to be fully processed.

It should be noted that both studies that reported N400 differences contrasted thematic and specific subtypes of taxonomic relations: vertical hierarchical relations (*offspring–grandson*) [21] and general function relations (*lamp–mirror*) [22]. This is important to have in mind when comparing results across studies, since taxonomic relations typically denote category co-members at the same hierarchical level (e.g. cow—donkey). Matching representations of *cow* and *donkey* may require different processing mechanisms than matching representations of *cow* and *animal* [23], and hence the size and timing of the N400 effect may differ.

Thus, although there is robust evidence for the behavioral distinction between thematic and taxonomic processing in the literature, studies reporting a neural distinction are still rare. We believe that detecting the expected N400 differences in thematic and taxonomic processing in the previous studies may have been undermined by task type, stimulus selection (e.g. sample size, restrictions as a choice of specific semantic subtypes) and the way N400 is quantified. Thus, we decided to follow the traditional definitions of thematic and taxonomic relations and base stimulus selection on the extensive stimulus sets of the previous studies showing behavioral differences in thematic and taxonomic processing [10, 24–28]. We compared thematic pairs, pairs that frequently co-occur and play complementary roles in the same scenario or event [6–7] and taxonomic pairs, pairs that denote objects belonging to the same superordinate category [6–7]. We avoided pairs that are both strongly thematically and taxonomically related (e.g. *cat*–*dog*). A large set of stimuli (70 match-thematic-taxonomic-unrelated quadruplets) and a repeated-measures design were used in order to boost the statistical power of the test and thus increase the chance to detect the expected effects.

Also, we used a simple task in which participants were not required to assess the relationship between item pairs, but thematic or taxonomic processing was hidden behind the main task which required processing both items in a pair in order to respond accurately. Thus we used a matching task in which both stimuli were available long enough to be easily perceived and participants only needed to answer whether two items were the same or different. This allowed for using match condition as a baseline, thus comparing thematic and taxonomic mismatches to the match, rather than only contrasting various kinds of mismatches (as in [20] for instance). In order to compare N400 effects across studies, it is important to take into account how the N400 effect was computed. Although in some previous studies N400 represented a difference in amplitude between the related and unrelated condition, in our study N400 was defined as a difference in amplitude between the match and the mismatch condition, as it was originally defined and computed by Kutas and Hillyard [29]. However, not all experimental designs allow for such a comparison, since there might be no “match” condition.

We predicted that both thematic and taxonomic relations would show an N400 effect, that is, that they would elicit more negativity than the match condition. Further, we expected thematic relations to elicit less negativity than taxonomic relations. This prediction is in accordance with the assumption that thematic relations are more salient than taxonomic relations [5–7] and supported by findings from the visual-world paradigm experiments and recent ERP findings [12–13, 22].

In order to avoid contamination of the stimulus-locked with the response-locked ERP components, we used an experimental design in which the button response is delayed (for discussion on this issue see, [30]). While delayed response design is recommended for experiments that aim to capture N400 effects, this design is not well suited for detecting behavioral effects of semantic priming. In behavioral semantic priming studies participants are usually asked to respond as quickly as possible in order to capture subtle differences in speed and accuracy of processing. Thus, an additional study, a behavioral version of the ERP experiment, was conducted in order to collect reliable reaction times. We did not make strong predictions regarding differences in accuracy and the speed of processing. Since accuracy in this kind of task should be high and since participants would have enough time to process both types of semantic information, the task may not be well suited for detecting subtle behavioral differences. However, if any difference between thematic and taxonomic processing would be observed, we would expect it to reflect an advantage for thematic pairs. The two studies used the same experimental design and stimuli, and the only difference was in the timing of the stimulus presentation.

## Experiment 1: Behavioral verification task

### Method

#### Ethics statement

This study was conducted in compliance with the guidelines and was approved by the Serbian Psychological Society Research Ethics Committee. All participants gave written informed consent prior to the study.

#### Participants

Nineteen adults, Serbian native speakers, were recruited to participate in this study. Participants were second-year psychology students participating for course credit.

#### Stimuli

The set of stimuli used consisted of 70 quadruplets. For each of the 70 target objects, one thematically, one taxonomically related, and one unrelated prime were selected. The testing stimuli consisted of 70 images of target objects and 280 Serbian prime-words denoting familiar objects’ labels of: (a) target objects (70), (b) thematically related objects (70), (c) taxonomically related objects (70), and (d) unrelated objects (70). Additional 140 picture-words pairs were used as fillers, to balance number of match and mismatch trials. The complete list of the stimuli is presented in S1 Table.

In order to validate stimulus selection, two groups of students who did not take part in the main study were asked to judge on a 7-point scale the strength of thematic (N = 16) and taxonomic (N = 19) relatedness of the stimulus pairs. Thematic pairs (M = 6.19, SD = .59) were judged as more strongly thematically related than taxonomic (M = 4.29, SD = .88) pairs (t(68) = 13.72, p < .001), and taxonomic pairs (M = 5.6, SD = .55) were judged more strongly taxonomically related than thematic (M = 4.42, SD = .70) pairs (t(68) = 11.43, p < .001).

Images were high-quality color photographs of real objects chosen from the Hemera image database [31], The Hatfield Image Test [32], and from commercial websites. All images were of the same size (300 x 220 pixels; 7.5° of visual angle) and all of them were presented within a white rectangle located in the center of a black background. Written words (primes) were presented in Serbian using the Latin alphabet, with black text (30 pixels high; 0.5° of visual angle) within a white rectangle located in the center of a black background. Participants were seated at a distance of 60 cm from a standard 15.6-inch monitor laptop computer used for the stimulus presentation.

#### Design

**Fig 1:**
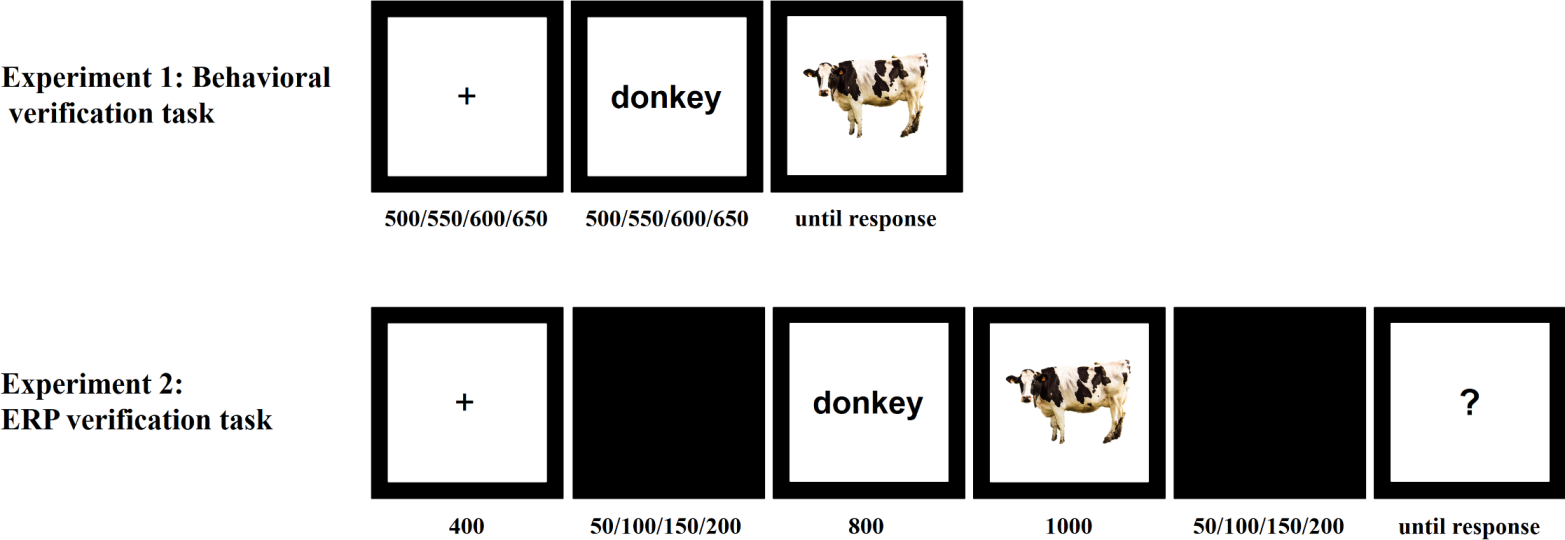
Structure of an experiment trial.

A within-participants design was used. There were four experimental conditions: (a) match, (b) thematic mismatch, (c) taxonomic mismatch, (d) unrelated mismatch. Trials consisted of pairs of words and images. Target objects were presented pictorially, and they were preceded by prime-word that labeled pictures correctly (match) or incorrectly (thematic mismatch, taxonomic mismatch or unrelated mismatch) (Fig 1).

Target objects were the same across all experimental conditions, thus allowing for the differences between experimental conditions to be attributed exclusively to the effects of the relationship type. Presentation order was randomized for each participant.

#### Procedure

Participants performed a label verification task. They were presented with word—image (prime—target) pairs and they were instructed to judge whether the word and image matched.

A typical trial started with the presentation of the fixation cross in the center of the screen for the jittered time range (500/550/600/650 ms) that varied from trial to trial. The fixation cross was followed by the presentation of the prime at the place of the fixation cross (for 500/550/600/650 ms). As soon as the prime disappeared, the centrally located target object photograph was presented. The target remained on the screen until the participant responded (Fig 1). We used Superlab 4.0 [33] for the presentation of the stimuli and data collection.

### Results

Accuracy analysis showed that the number of errors differed across the 4 conditions (χ^2^(3) = 118.75, p < .01). Participants responded most accurately in the unrelated mismatch condition (22 errors). In the match condition, participants made more errors than in the unrelated mismatch condition (64). Thematically (134) and taxonomically (152) related mismatch conditions provoked more errors than unrelated mismatch and match, but they were equally hard (p > .10).

**Fig 2:**
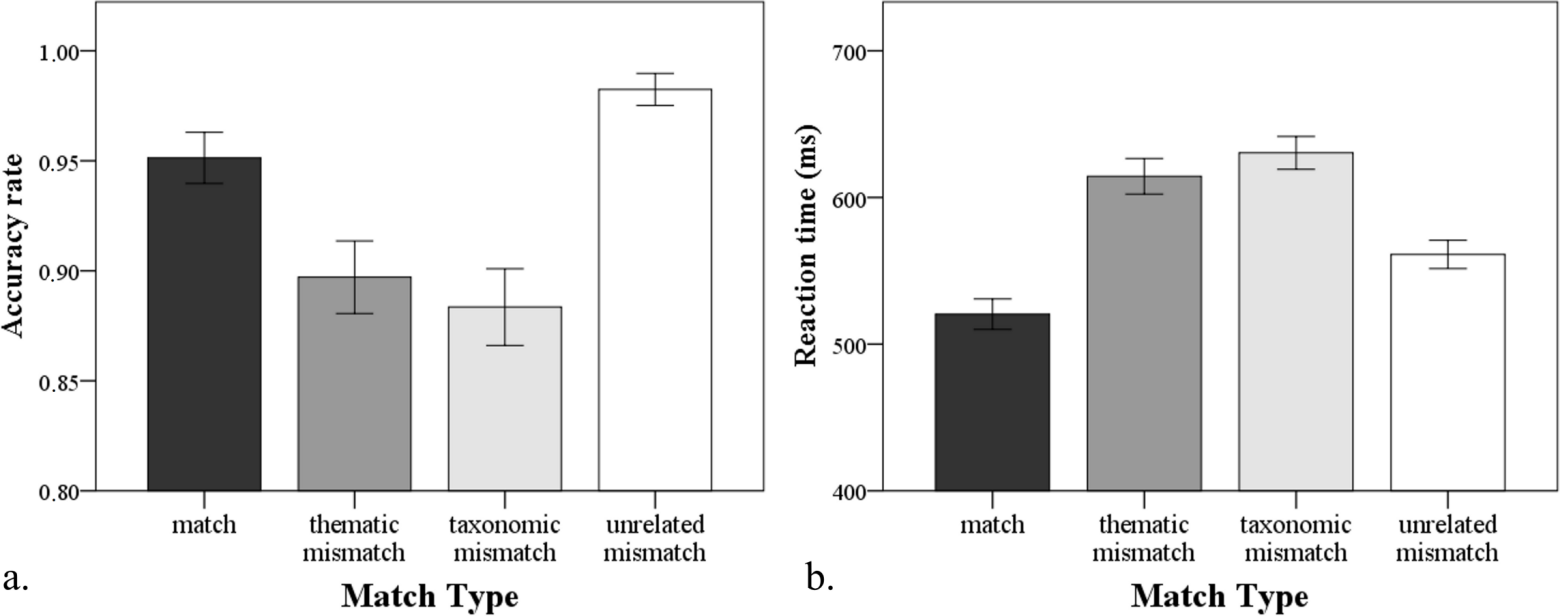
Accuracy rate (a) and average reaction time (b) across the four match types. Error bars represent confidence intervals.

In a preliminary analysis of reaction times, extremely long latencies (RT > 1500 ms) and extremely short latencies (RT < 100 ms) were excluded from the analysis. A repeated measures ANOVA with Match Type (match, thematic, taxonomic, and unrelated) as a within-subjects factor revealed a significant main effect of the Match Type (F(1.45, 24.62) = 28.17, p < .01, η^2^ = .62). Both types of related mismatch trials (thematic and taxonomic) were verified significantly slower (p < .01) than match trials and unrelated mismatch trials (p < .01), but there was no difference in the speed of rejecting unrelated mismatch and verifying match trials. There was no significant difference in speed between thematic and taxonomic mismatch trials (Fig 2).

## Experiment 2: ERP study

### Method

#### Ethics statement

This study was conducted in compliance with the guidelines and was approved by the Serbian Psychological Society Research Ethics Committee. All participants gave written informed consent prior to the study.

#### Participants

Twenty-four university students, Serbian native speakers, voluntarily participated in this study. Participants gave written informed consent prior to the study.

#### Procedure

The procedure was very similar to the one described for Experiment 1 (Fig 1). Participants were instructed to judge whether the word and image matched. Each trial started with a fixation cross (400 ms), followed by the blank screen (100 ms +/-50 ms jitter). Next, the prime word appeared (800 ms), immediately followed by the target image (1000 ms). After the image disappeared, the blank screen appeared again (100 ms +/-50 ms jitter) and was followed by the presentation of a question mark, which was the signal for participants to respond. The question mark remained on the screen until participant indicated whether the previously presented word-image pair was a match or a mismatch, by pressing C and N keys for matches and mismatches, using the index fingers of each hand. This allowed for participant response to be delayed in order to reduce the interference of motor responses in the EEG signal. Stimulus presentation was controlled by Superlab 4.0 [33].

#### Experimental setup for the ERP experiment

EEG signals were recorded continuously from the scalp in monopolar setup from 12 electrode sites located over left and right frontal (F3, F4), central (C3, C4), parietal-central (PC5, PC6), parietal (P3, P4), temporal (T5, T6), and occipital (O1, O2) areas. Electrodes were positioned according to the international 10–20 standard (Jasper, 1958). All electrodes were referenced to linked earlobes, and the ground electrode was positioned on the forehead. The EEG was amplified by a PSYLAB EEG8 biological amplifier in combination with PSYLAB SAM unit (Contact Precision Instruments, London, UK). Skin-electrode contact impedance levels were maintained below 5 kΩ. The signal was amplified (20k) and a 0.03–40 Hz hardware band-pass filter was applied. EEG was recorded continuously at a sampling rate of 500 Hz using NI USB-6212 (National Instruments, Austin TX) card for analog-to-digital signal conversion. For signals acquisition and online display, custom software with graphical user interface developed in LabVIEW 2010 was used (National Instruments, Austin, TX, USA) [34].

#### ERP processing

Offline signal processing was conducted using custom MATLAB routines (version 2010a, The Mathworks, Natick, MA, U.S.A.). A zero-phase 4th order Butterworth bandpass filter with 0.1–25 Hz cut-off frequencies was applied. The near-DC drift was filtered out by the high pass component, while muscle artefacts and 50 Hz noise, along with related harmonics, were removed by the low pass component. Individual 1000-ms epochs, which included 100 ms baseline period preceding and 900 ms interval following stimulus onset, were extracted from the ongoing EEG. All EEG channels were baseline corrected by subtracting the mean amplitude of 100-ms prestimulus interval from each epoch. Only trials without eye-movements and other artefacts whose absolute value of the signal from any of the channels did not exceed determined threshold were included in further analysis. Threshold was manually determined for each subject, and it ranged from 40–65 μV, with a mean value of 48.2 ± 7.1 μV. In order for participant data to be included in the further analysis, at least 60 trials from each experimental condition needed to be artefact-free. Data from two participants did not satisfy this criterion. For each participant and each condition at each electrode site, individual ERPs were calculated and segmented into 20-ms non-overlapping time bins. This resulted in 50 bins for which mean values were calculated. The first 5 bins represented the baseline period and the remaining 45 the period after the stimulus onset.

### Results

#### Behavioral results

Accuracy analysis showed that the number of errors differed across 4 conditions (χ^2^(3) = 52.60, p < .01). Accuracy was highest in the unrelated mismatch condition (8 errors) and participants made the most errors in thematic mismatch condition (73 errors). There was no difference between the match condition (45 errors) and the taxonomically (40 errors) related mismatch condition. Still, the average accuracy was extremely high (97.2%).

In a preliminary analysis of reaction times, extremely long latencies (RT > 1500 ms) and extremely short latencies (RT < 100 ms) were excluded from the analysis. The variation in timing due to jitter did not affect the effect of Match Type on response times and accuracy. A repeated measures ANOVA with Match Type (match, thematic, taxonomic, and unrelated) as a within-subjects factor revealed no significant effects regarding the reaction times.

#### ERP results: Statistical approach

**Fig 3:**
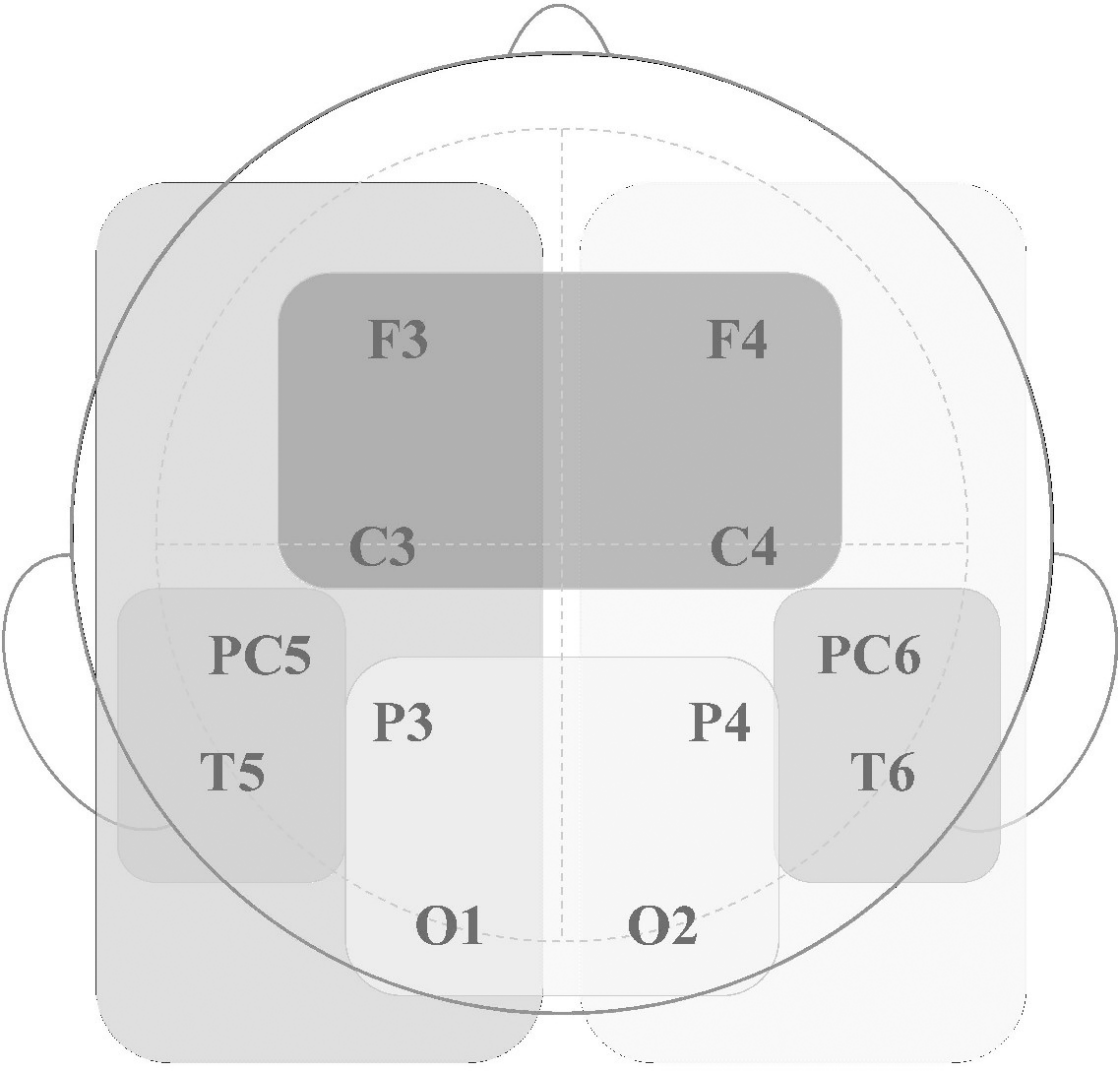
Layout of the electrode sites: Three bands, subdivided by hemisphere.

Data recorded from the 12 electrode sites were grouped into 6 zones—three bands, each subdivided into two lateral regions (left-right): fronto-central (F3, C3; F4, C4), temporal (T5, PC5; T6, PC6), and parieto-occipital (O1, P3; P4, O2) (Fig 3).

The order of analyses was as follows:

1. Instead of a priori selecting time windows based on the previous studies or basing the selection on visual inspection, a more fine-grained temporal analysis was used to better characterize the time-course of the N400 effects. We followed a procedure recommended by a number of previous ERP studies (e.g. [35, 36]). Namely, in the first step, repeated measures analyses of variance on mean amplitude ERP values in each 20 ms time window across six zones were performed. This allowed for the onsets and offsets of time windows of the effect of Match Type to be identified. Since in addition to N400, differences between relation types are occasionally reported in a later time window, P600 (e.g. [20]), we used the same procedure to detect onsets and offsets in this time window.
2. We identified two main time windows of interest. The first ranges from 280 ms to 460 ms (N400) and the second spreads from 520 ms to 600 ms (P600) post-stimulus interval. After time windows of interest were identified, mean amplitudes for each experimental condition were calculated.
3. Two 4x3x2 repeated measures ANOVAs with within-subjects factors of Match Type (match, thematic mismatch, taxonomic mismatch, and unrelated mismatch), Band (fronto-central, temporal and parieto-occipital), and Laterality (left and right) and mean amplitude as dependent measure were conducted.
4. Zone-by-zone repeated measures ANOVAs with Match Type as within-subjects factor were conducted for the time windows of interest.
5. In order to test for the latency effects, we tested differences between thematic and taxonomic mismatch N400 effect in the early N400 window ranging from 280 to 320 ms, and late N400 window ranging from 420 to 460 ms.

Greenhouse–Geissser corrections were applied where necessary.

#### Baseline

The experimental conditions did not differ during the baseline period. There was no effect of laterality; however, the effect of the band reached statistical significance (Band: F = (2, 28) = 4.90, p < .05, η = .26). Pairwise comparisons of Band levels did not reach significance.

#### Time window 280–460 ms

The ERP differed across the scalp (Band: F = (2, 28) = 27.33, p < .01, η = .66; Laterality: F(1, 14) = 7.37, p < .05, η = .35). Fronto-central region (more negativity) differed from temporal and parieto-occipital. There was less negativity in the left than in the right hemisphere.

**Fig 4:**
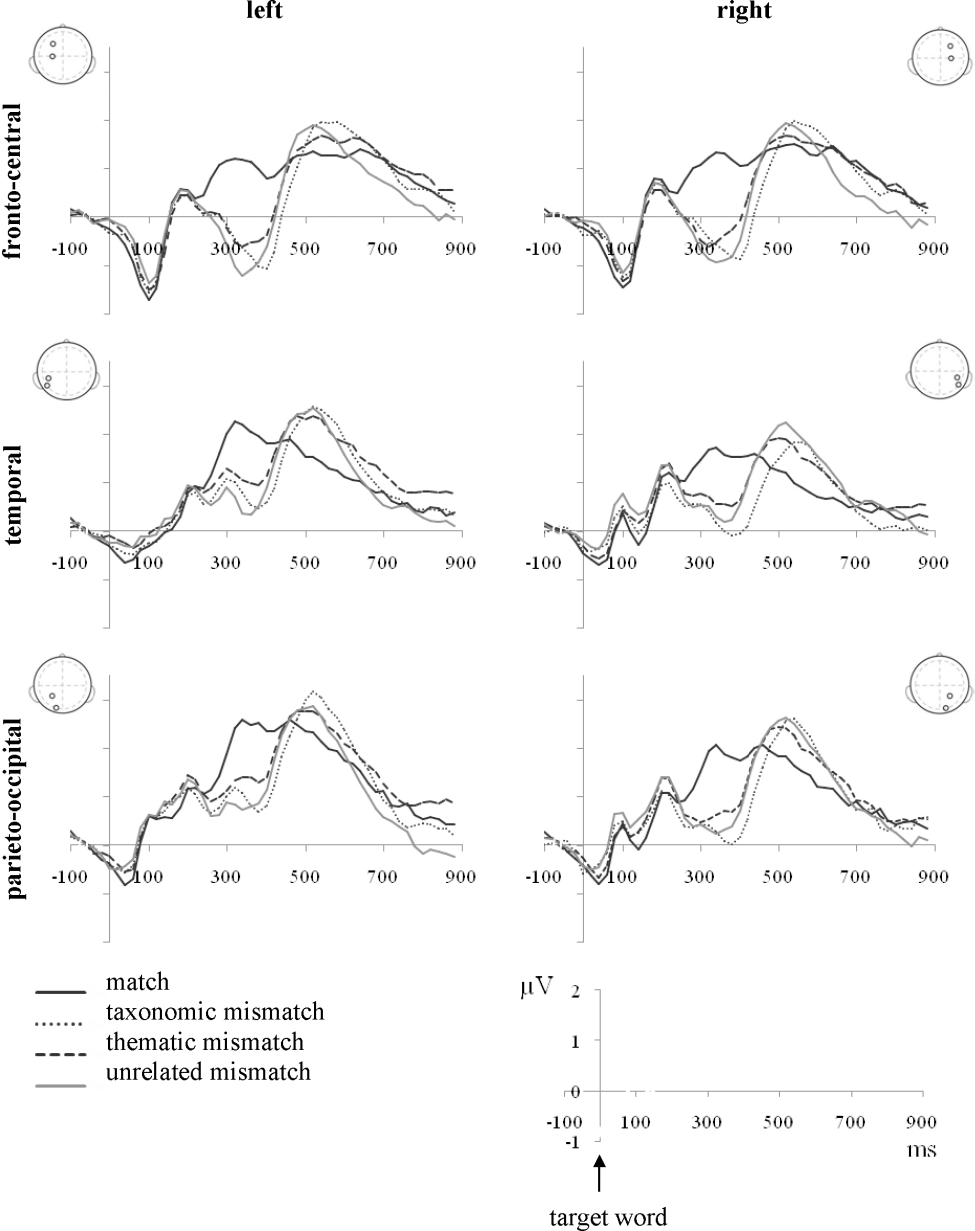
Average ERP waveforms time-locked to the presentation of targets in each of the four match type conditions for six zones.

**Fig 5:**
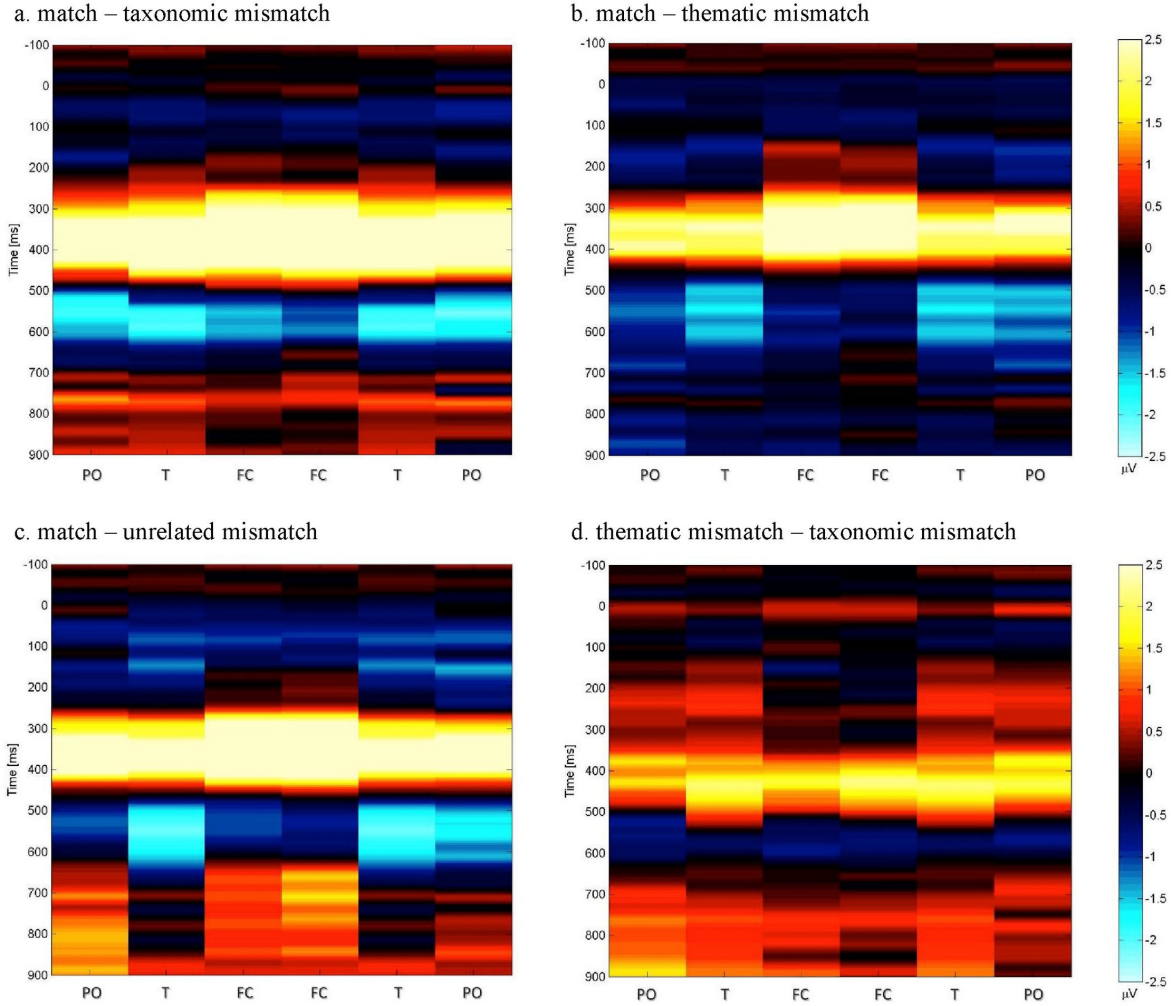
Dynamic maps showing the difference in ERP waves for match type conditions. (a) match–taxonomic mismatch (top left), (b) match–thematic mismatch (top right), (c) match–unrelated mismatch (bottom left), (d) thematic mismatch–taxonomic mismatch (bottom right). Time in milliseconds is shown on the vertical axis, starting in the baseline period 100 milliseconds prior to the onset of the critical stimuli. Labels on the horizontal axis stand for the six zones-of-grouping: P/0: parieto-occipital, T/PC: temporal, F/C: fronto-central. The color codes the value of the difference in the amplitudes of the waves. The scale on the left describes the difference in the amplitude of two waves in microvolts (μV).

In this window, the ERP differed significantly between match types (F(3, 42) = 17.01, p < .01, η = .55), with generally more negativity for mismatch than for match trials (p < .01). Differences between mismatch trials were not significant, except for a difference between thematic and taxonomic mismatch (p < .05) with more negativity for taxonomic mismatch. There were no significant interactions (Figs 4 and 5).

Zone-by–zone analysis revealed different patterns of effects over the six zones. Across the six zones, all three mismatch types elicited more negativity than match trials. In addition to the difference between mismatch and match trials, there was also a difference between thematic and taxonomic mismatch with more negativity for taxonomic mismatch in the right temporal (p < .01) and right parieto-occipital region (p = .06) (Table 1).

**Table 1:** Differences among match types across six zones in the N400 window (280–460 ms).

zone		ANOVA	Comparisons (Post-hoc, Bonferroni)
fronto-central	left	F_3, 54_ = 20.86, η = .54	match>mismatch^**^
	right	F_3, 57_ = 23.91, η = .56	match>mismatch^**^
temporal	left	F_3, 57_ = 14.88, η = .44	match>mismatch^**^
	right	F_3, 48_ = 16.26, η = .50	match>mismatch^**^; thematic>taxonomic^*^
parieto-occipital	left	F_3, 63_ = 14.40, η = .41	match>mismatch^**^
	right	F_3, 60_ = 19.93, η = .50	match>mismatch^**^; thematic>taxonomic^**‡**^

All F tests were significant on p < .01. Significance levels of Post-hoc tests are marked accordingly

^**^ p ≤ .01

^*^ p ≤ .05

^**‡**^ p = .06.

In addition to the difference in amplitude between the effects of thematic and taxonomic mismatch on N400, Fig 5 suggests latency effects (panels a and b). Statistical tests confirmed that in 280–320 ms time window thematic and taxonomic mismatch produced comparable mismatch effects, while in the late 420–460 ms window taxonomic items produced stronger mismatch effect than thematic pairs (Table 2).

**Table 2:** Latency effects—differences between thematic and taxonomic trials in early and late N400 window.

time window	planned contrasts (paired samples t tests, Cohen’s d)	
	match vs. taxonomic	match vs. thematic
280–320	t_118_ = 10.33, d = .95	t_118_ = 8.19, d = .75
420–460	t_118_ = 9.50, d = .87	t_118_ = 3.10, d = .28

All t tests were significant on p < .01 after corrected for the number of tests run.

#### Time window 520–600 ms

In the 520–600 ms time window, the ERP differed across the scalp (Band: F = (2, 28) = 5.79, p < .01, η = .29; Laterality: F(1, 14) = 5.90, p < .05, η = .30; Band x Laterality: F(2, 28) = 3.59, p < .05, η = .20) and it also differed according to Match Type (F(3, 42) = 5.34, p < .01, η = .28).

We found less positivity for match than for taxonomic mismatch (p < .01) and thematic mismatch (p < .05)–mirroring the effect detected in the earlier time window.

Zone-by-zone analysis uncovered that the effect of Match Type did not reach statistical significance in the fronto-central region (Table 3). There was only a marginally significant difference between taxonomic mismatch and match (p = .058) in the left fronto-central region.

**Table 3:** Differences among match types across six zones in P600 window (520–600 ms).

		ANOVA	Comparisons (Post-hoc, Bonferroni)
fronto- central	left	p > .05	match<taxonomic^**‡**^
	right	p > .05	
temporal	left	F_3, 57_ = 6.12, η = .24	match<taxonomic^**^; match<thematic^*^
	right	F_3, 48_ = 5.46, η = .25	match<taxonomic^*^; match<thematic^*^; match<unrelated^*^
parieto-occipital	left	F_3, 36_ = 3.51, η = .14	match<taxonomic^**^
	right	F_3, 60_ = 4.26, η = .18	match<taxonomic^*^

^**‡**^ p = .06

* p ≤ .05

** p ≤ .01.

In the temporal and parieto-occipital regions, match elicited less positivity than mismatched trials. In both temporal and parieto-occipital, left and right regions, there was a significant effect of the taxonomic mismatch. Additionally, thematic mismatch differed from match trials in left and right temporal region; and the difference between match and unrelated mismatch reached significance in right temporal region only.

## Discussion

The main goal of the studies presented in this paper was to examine the temporal dynamics and salience of thematic and taxonomic categories in tasks that minimize the influence of intentional, strategic processing.

Experiment 1 tested relationship type influence in an object verification task. Although related mismatches yielded more errors and took more time to be processed than unrelated mismatches and match trials, there was no significant difference in speed and accuracy between thematic and taxonomic trials. Behavioral results of the ERP study that used the same design, with the exception of the delay introduced between the target item presentation and the moment when participants were allowed to respond, were somewhat different. The null result in the analysis of the relationship type effect on response times could be expected, since delayed response obstructed the detection of subtle differences in the speed of processing. Regarding the accuracy, once again, participants responded most accurately in the unrelated condition. The match condition and taxonomic condition were equally hard, while participants made the most errors in thematic condition. One possible interpretation of the difference in accuracy across the conditions may be attributed to the difference in processing times. It is possible that prolonged response allowed for the integration of thematically related items to take place, which resulted in a higher number of false positives. It should be noted that accuracy was high in both experiments (94 and 98 percent, respectively), but that participants were even more careful in the ERP experiment, making more than three times fewer errors on average.

In the ERP study, semantic mismatches were followed by a negative component N400, in a time window between 280 and 460 milliseconds after the onset of the target. Concerning the processing of thematic and taxonomic relationships, which is more relevant to our question, analysis has revealed significant differences with more negativity for taxonomic mismatch. Zone by zone analysis showed that the difference between thematic and taxonomic trials was strongest in the right temporal (p < .01) and the right parieto-occipital region (p = .06), while in other regions there was a trend towards the same pattern but differences did not reach significance.

Less negativity for thematic mismatches would typically be taken as evidence of easier semantic integration of thematically related items, such as *cow* and *milk*, compared to taxonomically related items, such as *cow* and *donkey*. In other words, *milk* is a more appropriate semantic context for *cow*, than *donkey*. Some researchers also interpret the N400 component in terms of ease of accessing information from semantic memory (see [15]). Since the initial N400 article was published [29], it has been repeatedly shown that N400 is sensitive to the degree of semantic overlap, that is, to the similarity of the target item and the expected object or the perfect match (see [15]). Analogous to the priming effects, semantic similarity decreases response latencies and reduces N400 amplitude. In other words, search through semantic memory may be more efficient when using thematically related objects as cues or context, that is, the links in semantic memory may be stronger for thematically related compared to taxonomically related objects.

ERP differences between thematic and taxonomic relations were reported in two recent studies [21–22], which also found less negativity for thematic in comparison to taxonomic pairings. It is important to note that these studies used different subtypes of taxonomic relations (vertical hierarchical and general function relations) but obtained the same pattern of results as we did in our study where we contrasted thematically related objects and category co-members at the same level of hierarchy. Thus, our results complement previous studies by showing that the distinction between thematic and taxonomic relations holds across different relation subtypes. Furthermore, it offers a link with studies reporting behavior-based distinction by showing that the dissociation in the processing thematic and taxonomic relations extends to the lexical-semantic processing level.

As previously stated, most of the previous studies failed to find a difference in ERP responses of thematic and taxonomic types of relationships [17–20], at least when it comes to the differences during the N400 time window. We suggested that the failure to detect the differences may be due to the differences in how the N400 is computed. Similar to some previous studies (e.g. [19–20]), amplitudes of thematic and taxonomic responses were not significantly different from the amplitude of the unrelated response. In our study, robust N400 effects were found when comparing related and unrelated mismatch with match trials, but if we were to compare only the differences between the related and unrelated pairs, we would fail to detect the N400. However, there was a trend towards an attenuation of the N400 component for thematically related pairs. This trend was statistically detected in the difference between thematic and taxonomic mismatches.

Moreover, differences between thematic and taxonomic priming in our study were not only marked in amplitude, but also in latency of N400. Negativity elicited by thematic primes was significantly attenuated in the late N400 window (420–460 ms) compared to the effects produced by taxonomic primes. This result is in accordance with the results from visual world paradigm, which have shown that competition effects were longer lasting for taxonomic in comparison to thematic distractors [12–13]. Different time courses of activation of taxonomic in comparison to thematic information could also be expected following studies suggesting more effort is need to process categorical information [37–38].

Although we did not have strong predictions regarding components other than the N400, we detected differences in amplitude of the ERP wave in a later time window between 520 and 600 milliseconds. In this time window (P600), mismatch trials elicited less negativity than match trials. Taxonomic mismatch was different from the match condition across left fronto-central region, and temporal and parieto-occipital regions, while thematic mismatch was different from the match only across temporal regions. Similar results were obtained in a study by Chen et al [20], where larger P600 was reported for taxonomically related compared to unrelated and thematically related words (500–600 ms). Considering the fact that Chen et al [20] found differences between thematic and taxonomic relations in P600 window only, the authors interpreted this finding as a reflection of late semantic integration processes. However, the late positivity following an N400 is typically associated with the reprocessing or re-evaluation of the content based on the outcomes of prior syntactic and semantic analysis [39–40]. Larger P600 for taxonomically related items may indicate that taxonomic relations are less constraining than thematic relations and thus do not create a strong expectation of a specific following word. For the word *horse* as a cue, there are many comparably good taxonomic matches. Since most or all members of the horse family (e.g. donkeys, zebras, ponies), or more generally speaking, all category members at the same level of taxonomic hierarchy, have the same likelihood, the cognitive system may need to re-evaluate these other possibilities after initially processing and recognizing the existence of the relationship. On the other hand, groups of highly related thematic matches are more narrow (as a consequence of the differentiation in likelihood of specific thematic matches), which makes this condition more constrained. For example, for the word horse as a prime, saddle is the most likely thematically related word to follow, and there are only a few other competitors (e.g. jockey, reins). Alternatively, the strength of expectation of a following item may be higher in the thematic condition because thematic relations are often temporally oriented (e.g. A cause/produce B), which is not the case for taxonomic relations (e.g. A and B are members of the same category). A similar interpretation was offered by Chen at al [20], who argued that taxonomic pairs may be processed with less syntactic flow because they lack the external context, whereas thematic relations provide that context. Nevertheless, it is clear that these possibilities cannot be distinguished by the current experiment and that more research is needed to understand the exact mechanism by which the registered effects were generated.

The present study has shown that both thematic and taxonomic types of knowledge are decoded quickly, unintentionally, and that they both facilitate recognition of the succeeding information. Additionally, it provides evidence for differences in salience and timing of thematic and taxonomic knowledge activation, showing that the distinction long recognized in the psychological literature is also found in neuropsychological correlates of meaning integration and stimulus re-evaluation.

## Conclusion

Taken together, differences found between thematic and taxonomic conditions in our ERP study suggest easier processing of the thematic information during semantic integration phase (at around 400 ms after the stimulus onset) and a need for additional reprocessing of taxonomic information (at around 600 ms after stimulus onset).

So far, ERP differences in thematic and taxonomic processing have rarely been reported, with several studies reporting no differences [17–20] and a few studies reporting the similar pattern of results to the one obtained in our study [20–22]. Thus, this result is among the first to offer strong evidence for the neural distinction of thematic and taxonomic thinking in early phases of conceptual processing.

## Supporting information

S1 Table Complete list of stimuli: English translation of the Serbian words used in the study. (DOCX)
