# Correction: Comparing the temporal dynamics of thematic and taxonomic processing using event-related potentials

**DOI:** 10.1371/journal.pone.0218485

**Published:** 2019-06-11

**Authors:** Olivera Savic, Andrej M. Savic, Vanja Kovic

## Notice of republication

An incorrect version of Fig 1 was published in error. This article was republished on June 3, 2019 to correct for this error. Please download this article again to view the correct version.

## References

[1] Savic O, Savic AM, Kovic V (2017) Comparing the temporal dynamics of thematic and taxonomic processing using event-related potentials. PLoS ONE 12(12): e0189362 10.1371/journal.pone.0189362 29236767 PMC5728532

