# Supplementary material for: Comparing the temporal dynamics of thematic and taxonomic processing using event-related potentials
Source: PLoS One. 2017 Dec 13;12(12):e0189362. doi: 10.1371/journal.pone.0189362 (PMC5728532; doi:10.1371/journal.pone.0189362)
Supplement: S1 Table — (DOCX) [file pone.0189362.s001.docx]

Comparing the temporal dynamics of thematic and taxonomic processing using event-related potentials

Olivera Savic, Andrej Savic, Vanja Kovic

*** Correspondence:** Olivera Savic: [savic.7@osu.edu](mailto:savic.7@osu.edu)

S1 Table. Complete list of stimuli: English translation of the Serbian words used in the study.

|  | **target** | **prime type** | | | **filler** |
| --- | --- | --- | --- | --- | --- |
|  |  | **thematic** | **taxonomic** | **unrelated** |  |
| 1 | *horse* | *saddle* | *zebra* | *pin* | *painting* |
| 2 | *hen* | *egg* | *sparrow* | *rug* | *spade* |
| 3 | *cow* | *milk* | *donkey* | *blanket* | *rain* |
| 4 | *worm* | *apple* | *snail* | *keyboard* | *cross* |
| 5 | *chalk* | *blackboard* | *pencil* | *ladybug* | *razor* |
| 6 | *belt* | *pants* | *suspenders* | *telephone* | *fire* |
| 7 | *sparrow* | *nest* | *goose* | *chandelier* | *lantern* |
| 8 | *flower* | *vase* | *tree* | *fan* | *barrell* |
| 9 | *coconut* | *palm tree* | *pineapple* | *alarm* | *bottle* |
| 10 | *boat* | *oars* | *speedboat* | *cookie* | *onion* |
| 11 | *hammer* | *nail* | *pliers* | *step* | *crab* |
| 12 | *bee* | *honey* | *butterfly* | *balcony* | *kangooroo* |
| 13 | *pearl* | *shell* | *diamond* | *TV* | *tambour* |
| 14 | *brush* | *hair* | *comb* | *wheat* | *clarinet* |
| 15 | *aquarium* | *fish* | *cage* | *scales* | *gate* |
| 16 | *hedgehog* | *spines* | *bear* | *peacock* | *artichoke* |
| 17 | *spoon* | *soup* | *fork* | *shadow* | *tie* |
| 18 | *olive* | *oil* | *plum* | *socket* | *flowerpot* |
| 19 | *moon* | *sky* | *sun* | *sole* | *ottoman* |
| 20 | *ship* | *anchor* | *speedboat* | *hanger* | *cherry* |
| 21 | *pencil* | *eraser* | *pen* | *bathtub* | *slipper* |
| 22 | *key* | *lock* | *opener* | *bridge* | *timbrel* |
| 23 | *monkey* | *banana* | *giraffe* | *case* | *umbrella* |
| 24 | *scissors* | *paper* | *saw* | *bag* | *lamp* |
| 25 | *mouse* | *cheese* | *fox* | *camera* | *dolphin* |
| 26 | *dog* | *bone* | *leopard* | *calendar* | *duck* |
| 27 | *gold* | *bracelet* | *silver* | *tape recorder* | *eggplant* |
| 28 | *shoe* | *shoelace* | *boot* | *shelf* |  |
| 29 | *cannon* | *cannonball* | *pistol* | *sticker* | *penguin* |
| 30 | *elephant* | *peanuts* | *camel* | *box* | *seal* |
| 31 | *squirrel* | *hazelnut* | *kangaroo* | *pool* | *tigar* |
| 32 | *rabbit* | *carrot* | *lion* | *stone* | *puma* |
| 33 | *hand* | *watch* | *foot* | *leaf* | *drill* |
| 34 | *ball* | *beach* | *balloon* | *rasp* | *fife* |
| 35 | *sword* | *shield* | *spear* | *backyard* | *piano* |
| 36 | *bell* | *school* | *siren* | *garden* | *guitar* |
| 37 | *frog* | *swamp* | *lizard* | *steak* | *harp* |
| 38 | *hat* | *head* | *turban* | *blackberry* | *trumpet* |
| 39 | *frying pan* | *onion* | *pot* | *watermelon* | *violin* |
| 40 | *brooch* | *lapel* | *badge* | *paprika* | *cat* |
| 41 | *ring* | *hand* | *necklace* | *melon* | *firefly* |
| 42 | *sheep* | *wool* | *zebra* | *cucumber* | *dragonfly* |
| 43 | *castle* | *prince* | *cathedral* | *hammock* | *grasshopper* |
| 4 | *king* | *crown* | *president* | *perfume* | *triangle* |
| 45 | *purse* | *wallet* | *backpack* | *corn* | *lightbulb* |
| 46 | *crib* | *baby* | *chair* | *button* | *rattles* |
| 47 | *towel* | *soap* | *handkerchief* | *curtain* | *xylophone* |
| 48 | *butter* | *bread* | *cream cheese* | *eagle* | *sock* |
| 49 | *book* | *library* | *newspaper* | *pencil case* | *sweater* |
| 50 | *ear* | *earring* | *nose* | *pyjama* | *basket* |
| 51 | *door* | *house* | *window* | *baby powder* | *skirt* |
| 52 | *car* | *traffic lights* | *bus* | *frame* | *dress* |
| 53 | *cake* | *candles* | *pizza* | *rhinoceros* | *cutter* |
| 54 | *bed* | *pillow* | *table* | *wire* | *tomato* |
| 55 | *glass* | *straw* | *plate* | *grapes* | *grapes* |
| 56 | *sea* | *sand* | *lake* | *pot* | *fir tree* |
| 57 | *turtle* | *shell* | *alligator* | *grass* | *closet* |
| 58 | *pipe* | *tobacco* | *cigarette* | *folder* | *strawberry* |
| 59 | *feather* | *ink* | *fur* | *refrigerator* | *lighthouse* |
| 60 | *flag* | *race* | *banner* | *tea* | *grater* |
| 61 | *sponge* | *detergent* | *dishcloth* | *radish* | *radish* |
| 62 | *well* | *water* | *tap* | *skis* | *croissant* |
| 63 | *stork* | *chimney* | *seagull* | *sled* | *whisk* |
| 64 | *bench* | *park* | *couch* | *shoulder* | *pumpkin* |
| 65 | *bicycle* | *path* | *scooter* | *paw* | *beret* |
| 66 | *iron* | *shirt* | *mixer* | *bowl* | *mirror* |
| 67 | *church* | *cross* | *mosque* | *pie* | *saxophone* |
| 68 | *airplane* | *pilot* | *train* | *chocolate* | *scarf* |
| 69 | *coat* | *hanger* | *jacket* | *radiator* | *cauliflower* |
| 70 | *glasses* | *eyes* | *magnifier* | *quilt* | *headphones* |
